# Controversies in laparoscopic repair of incisional hernia

**DOI:** 10.4103/0972-9941.25670

**Published:** 2006-03

**Authors:** Abeezar I. Sarela

**Affiliations:** Consultant in Upper GI and Minimally Invasive Surgery, Hon. Senior Lecturer in Surgery, The General Infirmary at Leeds, The University of Leeds School of Medicine, Leeds LS1 3EX, UK

**Keywords:** Intra-peritoneal onlay mesh, ventral hernia repair, composite mesh, dual-sided mesh

## Abstract

**Background:**

Incisional hernias can be a significant problem after open abdominal surgery. Laparoscopic incisional hernia repair (LIHR) is conceptually appealing: a large, abdominal wall re-incision with potential wound-related ill effects is avoided and an intra-peritoneal onlay mesh is expected to provide security that is equivalent to open, retro-muscular mesh repair. As such, LIHR has gained substantial popularity despite sparse, randomised clinical data to compare with conventional, open repair.

**Aim:**

To enumerate and discuss important, controversial issues in patient-selection, technique and early post-operative care for LIHR.

**Materials and Methods:**

Pragmatic summary of comprehensive review of English language literature, discussion with experts and personal experience.

**Outcomes:**

Six important areas of some dispute were identified: 1. Size of abdominal-wall defect that is suitable for LIHR: Generally, defect-diameter > 10 cm is better served by open retromuscular repair with tension-free re-approximation of the edges of the defect. 2. Extent of adhesiolysis: Complete division of adhesions to the anterior abdominal wall may identify sub-clinical “Swiss-cheese” defects but incurs some risk of additional complications. 3. Intra-operative recognition of enterotomy: Possible options are either laparoscopic suture of bowel injury and simultaneous completion of LIHR, or staged LIHR or conversion to open suture-repair. 4. Choice of mesh: “Composite” meshes are regarded as the current standard of care but there is paucity of data regarding potential dangers of intra-peritoneal polypropylene mesh. 5. Technique of mesh-fixation: Trans-parietal sutures are more secure than tacks, with limited data to correlate with post-operative pain. 6. Alarm over post-operative pain: Unlike other advanced laparoscopic operations, the specificity of pain as a marker of intra-abdominal sepsis after LIHR remains unclear.

**Conclusion:**

Recognition of and attention to controversial issues will promote increased success of LIHR.

Laparoscopy has revolutionised the practise of surgery by imparting the ability to avoid major abdominal-wall incisions.[1,2] Thus, laparoscopic surgery is expected to reduce the burden of incisional hernias, but such morbidity of the era of conventional, open surgery is likely to remain a fairly frequent problem for the foreseeable future. It is well-established that repair of sizeable incisional hernias with a mesh, most commonly constructed of polypropylene, is associated with significantly reduced incidence of recurrent herniation as compared to suture-repair without mesh.[3] Also, the mechanical superiority of mesh-placement in the pre-peritoneal or retro-muscular space (sublay or underlay) over mesh that is fixed anterior to abdominal wall musculature (onlay) is conceptually apparent.[4,5] The seminal description of laparoscopic incisional hernia repair (LIHR) was published over 10 years ago and this technique involves an intra-peritoneal onlay mesh (IPOM) i.e. mesh-placement deeper by one abdominal-wall-layer than in the open sublay repair.

In common with most laparoscopic alternatives to open surgery, LIHR has gained sufficient popularity to be considered as a standard procedure, despite sparse systematic or randomised comparative data. The security or reliability of repair, measured by the incidence of recurrent herniation, in mainly retrospective, selected-institution series of open repair versus LIHR has been extensively reviewed.[6,7] Definitive comparison is difficult because of heterogeneity in case-mix and technique as well as length and accuracy of follow-up but, overall, LIHR appears to be at least as secure as open mesh repair; this impression is consistent with the similar prevalence of operations for recurrent incisional hernia before and after the introduction of LIHR, in a large population.[8] The well-established benefits of a minimally invasive approach, such as quick post-operative recovery and decreased risk of wound infection, favour the continuing increase in practise of LIHR. The aim of this paper is to discuss controversial issues in LIHR and to provide some potential solutions, based on personal experience, discussion with experts and comprehensive literature-review.

## Patient-selection

Are all patients, who require repair of incisional hernia, suitable for LIHR or are some patients better served by an open operation? Incisional hernias manifest an enormous spectrum of disease and, like any other operation, the capacity to successfully undertake LIHR, particularly in difficult situations, will largely depend on an individual surgeon's expertise. For example, technical difficulties, especially with laparoscopic adhesiolysis, may be anticipated in case of previous polypropylene mesh repair of the abdominal wall and in the morbidly obese.[9] Such patients are also likely to benefit most from a successful laparoscopic operation.[10] Nonetheless, limitations of LIHR, which may not be totally compensated by technical skill, need to be recognised. With an IPOM, the mechanism of repair is to bridge the hernia-defect without suturing the fascial tissues of abdominal wall. In contrast, for the open sublay-mesh repair, experts emphasise the importance of fascial re-approximation anterior to the mesh, with relief of tension (if necessary) by lateral release-incisions.[11] Such fascial closure is especially important in case of wide defects because of the risk that, in the absence of any overlying (anterior) support, the mesh may prolapse through the defect. The risk of prolapse may be reduced by ensuring that the mesh widely overlaps all edges of the defect, thereby diminishing outward-displacement pressure on the mesh-segment that bridges the defect. In the largest reported series of LIHR (850 patients), patients with subsequent recurrence had a mean defect-size of 184 cm^2^ and this was significantly larger than a mean defect-size of 124 cm^2^ for patients who did not suffer recurrence.[9] Thus, it may be prudent to offer open repair for particularly large ventral hernias. There are no objectively defined selection criteria but some experts have suggested limiting LIHR to cases where transverse separation of the fascial edges is ≤ 10 cm.

## Extent of adhesiolysis

Division of omental and bowel adhesions to the anterior abdominal wall is usually a necessary prelude to incisional hernia repair. In case of a small and clinically discrete hernia that is associated with a long laparotomy wound, is it necessary to divide adhesions to the wound beyond the extent that is required for the mesh to adequately overlap the edges of the fascial defect? A proposed advantage of LIHR, over open repair, is the ability to identify and repair clinically-unapparent “Swiss-cheese” defects, separate from the main hernia.[7] In most circumstances, it is sensible to perform a complete adhesiolysis and locate any such “Swiss-cheese” defects. However, there is paucity of data to support the notion that all untreated, sub-clinical fascial defects will progress to symptomatic disease. Thus, in selected cases (e.g. if extensive adhesiolysis is deemed to be particularly hazardous for enterotomy) it may be advisable to limit the dissection to the extent necessary for repair of the main hernia.

## Intra-operative recognition of enterotomy

An inadvertent enterotomy is a serious complication of laparoscopic adhesiolysis. Intra-operative recognition of an enterotomy is undoubtedly preferable to a “missed” injury but, although laparoscopic bowel-repair is usually feasible, peritoneal exposure to intestinal content raises significant concern of infection of an intra-peritoneal mesh. What are the strategies available to deal with such a situation? One option, which is particularly attractive when there is no enteric spillage, is to laparoscopically suture the perforation and proceed with IPOM, in conjunction with copious saline-lavage of the peritoneal cavity and intravenous antibiotics. A second option is to complete laparoscopic adhesiolysis and repair the bowel injury but to delay mesh-placement (i.e. perform a “staged repair”, within a fairly short interval), in order to optimise bacterial clearance and minimize the risk of infection. A similar approach is attractive if adhesiolysis has been difficult and there is high index of suspicion for a missed enterotomy; a planned re-laparoscopy after 24–48 hours provides the opportunity to confidently exclude a missed injury and to simultaneously place a mesh. In a large series, mesh infection was not encountered in any of 10 patients with laparoscopic repair of small intestinal injury and simultaneous or staged LIHR.[9] Similarly, in another series, there is no report of mesh infection in any of eight patients with laparoscopic repair of small bowel perforation and simultaneous LIHR.[12] Colonic injury is a more serious concern; there is no substantial evidence-base to guide decision-making. There are anecdotal reports of laparoscopic colonic repair with simultaneous IPOM, followed by either no evident mesh infection[9] or, alternatively, serious complications.[13] The author has experience of delayed presentation of mesh infection, six months after the patient made an uneventful recovery following laparoscopic repair of colonic perforation during LIHR. The optimal strategy in case of enteric injury needs to be decided on a case-by-case basis. A safe option, particularly if laparotomy has been undertaken because of the bowel injury, is to perform simply a suture-repair of the hernia and accept that the risk of mesh infection has been exchanged for a higher risk of hernia recurrence.

## Choice of mesh

This may be the most contentious issue in LIHR, particularly when financial cost is a major consideration. Incisional hernioplasty with polypropylene mesh was initially described in 1958;[14] subsequently, polypropylene (Prolene^®^, Marlex^®^, Trelex^®^) has been a popular material for open mesh repair. Polyester (Mersilene^®^, Dacron^®^) and expanded polytetrafluoroethylene (ePTFE, Gore-Tex^®^) have also been frequently used. In comparison to open repair, the paradigm-shift with LIHR is intra-peritoneal mesh placement with potential for direct contact between mesh and bowel. It has been well-demonstrated in animal models that the conventional meshes incite an inflammatory reaction and adhesions, which are least with ePTFE.[15] Conversely, polypropylene and polyester are better incorporated than ePTFE into tissues of abdominal wall and this is related to restricted tissue growth into the small pores (17–22 μm) of the ePTFE. Particularly with polypropylene, there is enormous concern, to the extent of contra-indication, about intra-peritoneal usage because of the risk of dense adhesions that involve bowel and may progress to intestinal obstruction or erosion. Most surgeons can recount anecdotes of serious intestinal complications following polypropylene mesh repair; this issue appears to be specifically examined in only one series, which comprised 136 patients with open polypropylene mesh repair.[16] In this latter series, the mesh was placed in the retromuscular position in 80% of patients and in 40% it was not possible to either close peritoneum or interpose omentum between mesh and bowel. Importantly, with median follow-up of three years, there was no case of intestinal obstruction or entero-cutaneous fistula. It seems reasonable to conclude that there is low risk of intestinal complications from intra-peritoneal polypropylene mesh, although long-term, population-based data are not available. A further concern is that dense adhesions, even if asymptomatic, can cause significant technical difficulty and possible adverse outcomes, if subsequent abdominal surgery becomes necessary. The widely accepted alternative to “simple” polypropylene is a “composite” or “dual-sided” mesh that offers a conventional material, such as polypropylene or polyester, in contact with the abdominal wall and an inert, potentially adhesion-resistant substance, such as collagen or cellulose, to interface with bowel. In experimental studies in rodents, about one-half the surface area of intra-peritoneal polypropylene mesh was adherent to bowel at 30 days after operation in 50% of cases as compared with no bowel adhesions to composite meshes.[17] The author has experience of Parietex^®^ (Sofradim/Tyco^®^; polyester-collagen), Proceed^®^ (Johnson & Johnson^®^; polypropylene-polydioxanone-oxidised regenerated cellulose) and Intramesh (Cousin^®^; Polypropylene-ePTFE). There are specific issues with each product; for example, with Parietex^®^, the collagen film is fragile and easily damaged; with Proceed^®^, recent reports of layer-separation have resulted in product-withdrawal; Intramesh^®^ is brittle and prone to laceration on suturing. Other popular products are Composix^®^ (Bard^®^; polypropylene-ePTFE), Sepramesh^®^ (polypropylene-Seprafilm^®^) and Dualmesh^®^ (Gore^®^; two layers of ePTFE with different pore-sizes: the visceral surface has 3 μm interstices that inhibit penetration by fibroblasts and the parietal surface has 22 μm interstices to promote tissue incorporation). Dualmesh^®^ differs from the original Gore-Tex Soft Tissue Patch^®^, which is a single layer of ePTFE (pore-size, 17–22 μm) that may attract bowel-adhesions. There is also much interest in slowly resorbable materials, such as Surgisis Gold^®^ (Cook^®^ collagen-glycosaminoglycan from porcine intestinal submucosa) and the European Hernia Association is currently running a randomised clinical trial that compares laparoscopic or open ventral hernia repair with Surgisis Gold^®^ versus nonresorbable material.[18] The financial-cost to clinical-benefit ratio for use of the substantially-expensive composite meshes is unquantified and is likely to remain as such because, given widespread acceptance of composite products, a randomised, clinical comparison with simple polypropylene mesh is unlikely to occur. In selected circumstances, it may be acceptable to use a simple mesh, if this can be completely excluded from bowel by interposition of omentum, but a composite mesh should be considered as the current standard of care.

## Technique of mesh-fixation

The mesh can be fixed to the abdominal wall using either solely tacks, of which a variety is available (e.g. Protac^®^, Endoanchor^®^), or a combination of sutures and tacks or only sutures. Two important outcomes that should guide the choice of fixation strategy are security of fixation and post-operative pain. The overall incidence of recurrence appears to be similar in series that employed tacks only versus those that used sutures.[7] Such data need to be interpreted with caution, recognising caveats about retrospective, non-randomised comparison. Conceptually, a 4 mm-long tack (Protac^®^) would be expected to penetrate only 2 mm into the abdominal wall, after allowing 1 mm for the thickness of the mesh and another 1 mm for the tack-profile that projects on the surface of the mesh. Thus, in obese patients, the tack may be restricted to the extra-peritoneal fat without purchase into muscle. At least one experimental study has demonstrated that the tensile strength of trans-parietal sutures is up to 2.5 times greater than that of tacks.[19] This latter study also showed that about 2 cm was the optimal distance between adjacent fixation points. Reluctance to use multiple trans-parietal sutures is largely related to a perceived association with increased post-operative pain, perhaps due to muscular ischaemia or entrapment neuropathy. Conversely, the authors' group noted severe, early post-operative pain with the use of a large number of tacks and substantial improvement in post-operative discomfort following conversion to a policy to use mainly sutures. It has been suggested that a delayed-absorbable suture-material, such as PDS, may potentially release entrapped nerves in due course and also permit mesh shrinkage without distortion of the abdominal wall. The sutures are, in any case, dispensable after fibrous incorporation of the mesh.

## Alarm over post-operative pain

Advanced laparoscopic surgery is generally associated with minimal post-operative pain and it is usual to anticipate a comfortable patient at 24 hours after operation. In contrast, substantial, early post-operative abdominal pain is a fairly regular feature of LIHR. Generally, excessive pain after laparoscopic surgery is a sensitive indicator of peritoneal inflammation and, in such circumstances, liberal use of early re-laparoscopy is valuable to confidently detect (or exclude) any serious intra-abdominal complication.[20] Since “missed” enterotomy is a grave concern in LIHR, particularly after a difficult adhesiolysis, correct interpretation of the significance of post-operative pain is an important issue. Whether or not to re-laparoscope a patient, who experiences severe post-LIHR pain, remains a clinical dilemma. There is no evidence-base to guide this issue, but it is apparent that the specificity of pain as a marker of bowel injury is lower after LIHR as compared with other laparoscopic procedures.
